# Poly(3-hydroxybutyrate-co-3-hydroxyhexanoate) Bionanocomposites with Crystalline Nanocellulose and Graphene Oxide: Experimental Results and Support Vector Machine Modeling

**DOI:** 10.3390/polym15183746

**Published:** 2023-09-13

**Authors:** Elizabeth Champa-Bujaico, Ana M. Díez-Pascual, Pilar Garcia-Diaz

**Affiliations:** 1 Universidad de Alcalá, Departamento de Teoría de la Señal y Comunicaciones, Ctra. Madrid-Barcelona Km. 33.6, 28805 Alcalá de Henares, Madrid, Spain; elizabeth.champa@uah.es (E.C.-B.); pilar.garcia@uah.es (P.G.-D.); 2 Universidad de Alcalá, Facultad de Ciencias, Departamento de Química Analítica, Química Física e Ingeniería Química, Ctra. Madrid-Barcelona, Km. 33.6, 28805 Alcalá de Henares, Madrid, Spain

**Keywords:** polyhydroxyalcanoate, bionanocomposites, graphene oxide, nanocellulose, mechanical properties, machine learning

## Abstract

Poly(3-hydroxybutyrate-co-3-hydroxyhexanoate) (PHBHHx) is a biodegradable and biocompatible bacterial copolymer used in the biomedical and food industries. However, it displays low stiffness and strength for certain applications. This issue can be solved via reinforcement with nanofillers. In this work, PHBHHx-based bionanocomposites reinforced with different loadings of crystalline nanocellulose (CNC) and graphene oxide (GO) were developed by a green and straightforward solution casting technique. Their crystalline nature and surface topography were explored via X-ray diffraction (XRD) and field-emission scanning electron microscopy (FE-SEM), respectively, their composition was corroborated via Fourier-transformed infrared spectroscopy (FTIR), and their crystallization and melting behavior were determined via differential scanning calorimetry (DSC). The nanofillers had a nucleating role, raising the crystallization temperature of the polymer, whilst hardly any changes were found in the melting temperature. Further, significant enhancements in the stiffness, strength, and thermal stability of the PHBHHx matrix were observed with the incorporation of both nanofillers, which was attributed to a synergic effect. The mechanical properties for various concentrations of CNC and GO were accurately predicted using a machine learning (ML) model in the form of a support vector machine (SVM). The model performance was evaluated in terms of the mean absolute error (MAE), the mean square error (MSE), and the correlation coefficient (*R*^2^). These bio-based nanocomposites are a valuable alternative to conventional petroleum-based synthetic polymeric materials used nowadays for biomedicine and food packaging applications.

## 1. Introduction

**Scheme 1:**
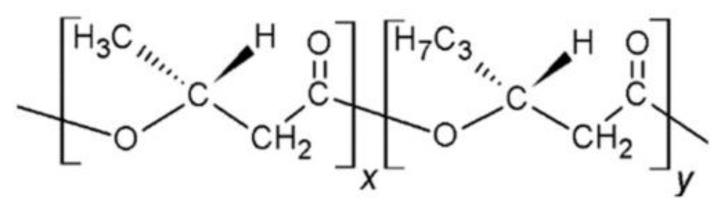
Representation of the structure of the PHBHHx copolymer.

Poly(3-hydroxybutyrate-co-3-hydroxyhexanoate) (PHBHHx) is a bacterial copolyester that corresponds to the polyhydroxyalkanoate (PHA) family [1]. It is a copolymer composed of hydroxybutyrate and hydroxyhexanoate units (Scheme 1), and its properties depend on the composition. Compared with other members of this family, such as poly(3-hydroxybutyrate) (PHB) and poly(3-hydroxybutyrate-co-3-hydroxyvalerate) (PHBV), PHBHHx has improved mechanical performance and a faster biodegradation rate [2]. Further, it has excellent biocompatibility with numerous cells like osteoblasts [3], fibroblasts [4], and chondrocytes [5]. Moreover, PHBHHx is less crystalline and has a higher melt viscosity, which is a desirable property for extrusion. PHBHHx films have been developed using solvent casting, electrospinning, and compression molding methods [6]. Nonetheless, the uses of PHBHHx have been restricted due to drawbacks including strong hydrophobic character and slow speed of crystallization. To solve these issues, lots of efforts have been focused on tailoring the properties of this copolymer and designing novel derivatives [7,8,9]. Better mechanical characteristics and thermal stability have been attained with the addition of low quantities of nanoparticles [7] or polymer blending [8,9], though the improvements are not enough for industrial applications. Additionally, literature articles related to PHBHHx nanocomposites are very scarce.

Graphene oxide (GO) is a 2D graphene derivative prepared via oxidation routes with a wide number of oxygenated groups on its surface, including COOH on the edges of the nanomaterial sheets as well as -OH and -O- groups on both sides of the sheet planes [10]. It is easily processed in an aqueous medium via ultrasonication and presents numerous benefits such as amphiphilicity, biocompatibility, outstanding thermal and mechanical properties, huge surface area, and antibacterial activity. However, traditional routes to prepare GO, such as Hummers’ method, are tedious and need noxious organic solvents. To solve this issue, a green and inexpensive method for the electrochemical preparation of GO was reported in a previous work [11], which enables us to carefully tailor the degree of nanomaterial oxidation and exfoliation via changing the electrochemical parameters. 

On the other hand, nanocellulose is regarded as a green material due to its high degradation rate, biocompatibility, and easy availability. It is an inexpensive and inexhaustible nanomaterial that occurs naturally in several structures, i.e., nanofibrillated cellulose (NFC) and crystalline nanocellulose (CNC), also known as cellulose nanocrystals and cellulose nanowhiskers (CNWs) [12]. Nanocellulose can be used to reinforce polymers, leading to green bionanocomposites with outstanding improvements in material performance compared with the raw matrices or traditional microcomposites. These improvements are ascribed to the outstanding properties of cellulose fibers such as superior stiffness and strength, excellent barrier properties, as well as ease of functionalization via chemical reactions.

In order to attain improved properties, different nanofillers can be incorporated into a polymeric matrix, leading to a hybrid or multifunctional nanocomposite with enhanced performance owing to a synergic effect [13]. In this regard, graphene oxide/nanocellulose hybrids have been combined and applied as nanoreinforcement in a chitosan matrix [14]. However, the preparation and characterization of hybrid multiscale nanocomposites are still in their infancy, and significant studies are required to obtain knowledge about how the combination of several fillers at the nanoscale can tune the matrix properties via interactions between the different nanocomposite constituents. In this work, multiscale PHBHHx-based nanocomposites comprising various loadings of CNC and GO were prepared via an easy, cheap, and environmentally friendly solution casting method. By adjusting the contents of both nanoreinforcements, the stiffness, strength, and thermal stability can be finely controlled for pursued applications. Further, the experimental mechanical properties were predicted via a machine learning (ML) algorithm. The ML model applied is comprehensively described in the following section. Predicting the mechanical performance of multiscale nanocomposites is crucial for cost-effective design with the aim to apply them in fields like food packaging or medicine. The results demonstrate that ML algorithms can accurately predict the mechanical characteristics of hybrid composites.

## 2. Methodology

The ML model applied herein is a support vector machine (SVM), which has been commonly applied in the scientific literature [15]. It was selected since it is one of the most common supervised learning algorithms applied to solve regression and real classification tasks owing to its potential to simulate non-linear relations between input and output variables. Therefore, it is highly valuable in the case of multiscale nanocomposites since their properties are influenced by numerous parameters. Besides, it is versatile and has good predictive accuracy [16]. The main goal of SVM in regression problems is to obtain a function that maps the input data to a space of higher dimensionality, where an optimal hyperplane can be drawn that separates the data points as well as possible [17], minimizing the prediction error and allowing a controlled margin of error for data points that cannot be perfectly separated [18].

To achieve an optimal model, the following hyperparameters must be set: (1) The regularization parameter, C, regulates the balance between the model complexity and its skill for optimally fitting the training data [19]. (2) The kernel function is responsible for the transformation of the input values into a larger dimensional space. This parameter determines how the similarity between data points in that transformed space is calculated. There are several common kernel types, such as linear, polynomial, and radial basis (RBF) kernels [20]. In this study, the RBF was applied, which is used when the variables have a nonlinear relation. (3) The gamma parameter indicates how far the effect of a single training example reaches. Thus, a low C value indicates “far” while a high value indicates “near”. It has a substantial influence on the model accuracy [21]. (4) The epsilon controls the margin around the regression hyperplane and affects the trade-off between the model fit and the acceptable margin of error.

SVM uses a combination of values of the abovementioned hyperparameters for each model, allowing it to learn optimally from the data. SVM was executed herein with the programming language Python V.3.9.6 and the library Scikit-learn v.0.23.2.

In this work, the prediction of the mechanical properties was carried out using the support vector regression (SVR) algorithm in combination with the hyperparameter search technique RandomSearchCV, in which the values of C and epsilon are varied.

The performance of the developed model was evaluated using 3 statistical indicators: The mean square error (MSE), which is determined as indicated in Equation (1):

(1) $$MSE=\frac{1}{n}\sum (y^{\prime }-y{)}^{2}$$

with *y*′ − *y* being the difference between each predicted and experimental value and *n* being the sample size.

On the other hand, the mean absolute error (MAE) is calculated as shown in Equation (2):

(2) $$MAE=\frac{1}{n}\sum \left|y^{\prime }-y\right|$$

MAE is less influenced by outliers than MSE since it is an arithmetic average of absolute errors. However,, MAE can underestimate the effect of big errors.

The coefficient of determination (*R*^2^) shows how correlated the predicted and measured values are and is estimated as indicated in Equation (3):

(3) $$R^{2}={\left(\frac{n(\sum yy\prime )-(\sum y)(\sum y\prime )}{\sqrt{\left[n\sum y^{2}-(\sum y{)}^{2}\right][n\sum {y^{\prime }}^{2}-(\sum {y\prime )}^{2}]}}\right)}^{2}$$

The closer *R*^2^ is to 1.0, the better the accuracy of the model.

## 3. Experimental Section

### 3.1. Materials 

The neat copolymer PHBHHx, comprising 12% HHx (M_w_ = 450,000; T_g_ = 2–10 °C, T_m_ = 150 °C), was provided by P&G (Cincinnati, OH, USA). A 0.1 mm thick high-density flexible graphite foil (HDFGF, C > 99.5%) was purchased from Beyond Materials, Inc. (Tucson, AZ, USA) and dried in an oven for 2 days prior to use. Ethanol, sulfuric acid (>99.9%), citric acid (C_6_H_8_O_7_, >99%), hydrogen chloride (36–38%), and microcrystalline cellulose (C_6_H_10_O_5_)_n_ in powder form (average size 20 µm) were purchased from Merck (Madrid, Spain). All the reagents were used as delivered without further purification.

### 3.2. Nanocomposite Preparation 

CNC was synthesized via hydrolysis of microcrystalline cellulose with hydrogen chloride and citric acid mixtures, as reported in a preceding study [22]. FE-SEM and TEM images of the as-synthesized CNC revealed that they show a rod-like morphology (Figure S1a,b) with a length in the range of 200–350 nm, a diameter of 8–15 nm, and an average aspect ratio (L/D) of 23.

GO was electrochemically synthesized at RT starting from raw HDFGF through a two-step procedure, as reported earlier [11]. An amount of 2 V was firstly applied for 10 min, followed by 1 min under 20 V. H_2_SO_4_ (98 wt%) was used as an electrolyte. After purification by several washing and centrifugation stages, the resulting GO was stored in dark conditions until further use. According to elemental analysis, its C/O ratio was 1.46 [11]. FE-SEM and TEM images of raw GO (Figure S1c,d) show a coarse surface topography with exfoliated and well-separated wrinkled graphene sheets, with thicknesses ranging from 30 to 10 nm. 

**Scheme 2:**
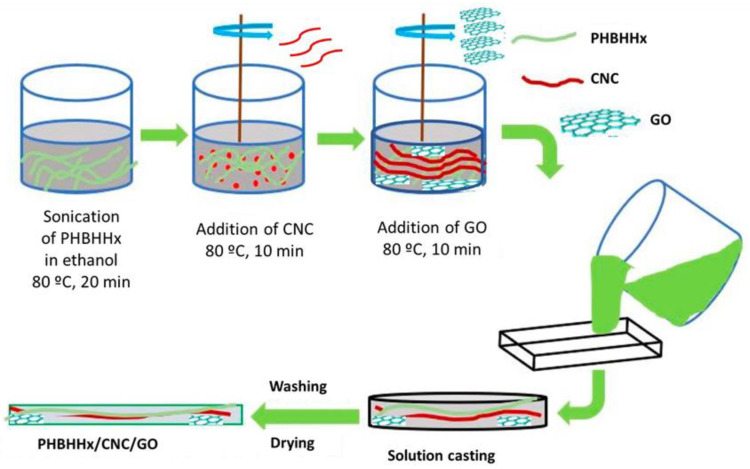
Depiction of the fabrication of ternary PHBHHx/CNC/GO hybrid composites.

The hybrid composites were fabricated using a simple solution casting process. Initially, PHBHHx was dissolved in ethanol via ultrasonication at 80 °C for 20 min. Then, the required amount of CNC was added and the blend was subjected to sonication for another 10 min under heating. Subsequently, the necessary amount of GO was added and sonicated for 10 min to reach a homogenous mixture, which was then poured into a Petri plate, washed with water, and subsequently dried for 48 in a vacuum oven. A representation of the nanocomposite manufacture is depicted in Scheme 2. For the sake of comparison, binary nanocomposites comprising only CNC or GO in the range of 0.5–2.5 wt% were also manufactured in a similar way. Photographs of the nanocomposites are shown in Figure S2.

### 3.3. Characterization 

The surface topography of the nanocomposites was explored via field-emission scanning electron microscopy (FE-SEM) with a JSM-IT800 Schottky microscope (JEOL, Peabody, MA, USA), operating at 10 kV. Prior to observation, the nanocomposite films were cryo-fractured via immersion in liquid nitrogen and afterward coated with a 5 nm gold overlayer to prevent charging during the experiments.

A PerkinElmer Spectrum 3 FT-IR spectrometer (PerkinElmer Inc., Shelton, CT, USA) equipped with an ATR sampling probe and a laser source at a wavelength of 633 nm was used to obtain the IR spectrum of the samples. Spectra were acquired at 25 °C, at a power of 1 mW, in the wavenumber range 500–4000 cm^−1^. At least five scans were recorded for each composite to reduce the signal-to-noise ratio.

X-ray diffraction (XRD) experiments were performed with a D8 Advance X-ray diffractometer (Bruker, Germany) equipped with a Cu Kα anode (λ = 0.1542 nm) operating at 40 kV and 30 mA. The patterns were acquired at room temperature over the 2Ɵ range of 5–60°, with a step size of 0.05°.

Thermogravimetric analysis (TGA) measurements were performed at a heating rate of 10 °C/min, in an inert environment, with a Q500 thermobalance (TA Instruments Ltd., New Castle, DE, USA). The experiments were carried out with a nitrogen flow rate of 50 mL/min. To remove moisture, the composites were dried for 24 h under vacuum prior to the measurements. 

A DSC 822e equipment (Metler Toledo GmbH, Greifensee, Switzerland) was used to determine the melting and crystallization temperature of the samples. Experiments were performed in an enclosed aluminum vessel with a N_2_ flow rate set at 50 mL/min. Composites were heated from 0 to 190 °C using a 10 °C/min ramp rate and then cooled down to RT.

Tensile properties were assessed with an 858 Mini Bionix testing device (MTS Systems Corporation, Eden Prairie, MN, USA) at 23 °C and 50% RH, with a crosshead rate of 1 mm/min, as specified in the ASTM D 638-03 standard. Prior to the measurements, the samples were acclimated for 1 day. Six specimens for each nanocomposite were measured to ensure reproducibility, and the average value was recorded.

## 4. Results and Discussion 

### 4.1. Surface Topography of PHBHHx/CNC/GO Nanocomposites

**Figure 1:**
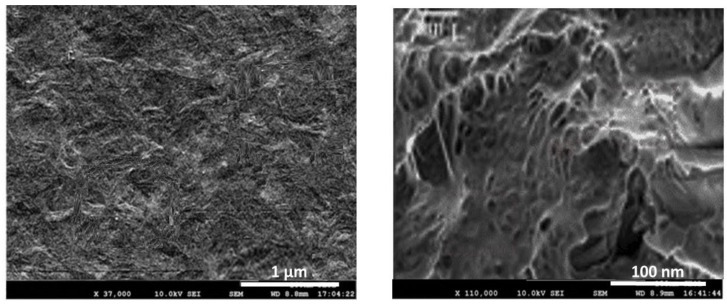
FE-SEM micrographs of the surfaces of the PHBHHx/CNC (1.0 wt%)/GO (1.5 wt%) nanocomposite at different magnifications.

FE-SEM was used to assess the surface topography of the multiscale PHBHHx-based nanocomposites and obtain qualitative information about the dispersion state of both nanofillers within the matrix. Representative micrographs of the cross section of the composite with 1.0 wt% CNC and 1.5 wt% GO, at two amplifications, are displayed in Figure 1. Analogous micrographs were found for the other ternary nanocomposites.

**Scheme 3:**
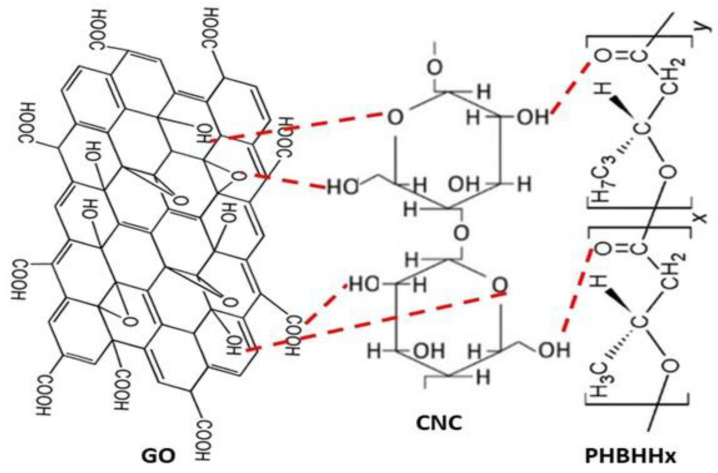
Schematic illustration of the H-bonding interactions between PHBHHx, CNC, and GO.

The nanocomposites show a porous, relatively coarse, and wavy surface topography made of GO nanosheets and CNC nanocrystals surrounded by a semicrystalline continuous phase of PHBHHx. The rigid white dots likely correspond to the rod-like CNC crystals, whilst the most flexible and fluffy ones are probably the 2D GO sheets. In the image at higher magnification, both nanofillers are found to be well dispersed throughout the matrix, randomly but evenly, forming a condensed, entangled, and intercalated network, likely due to the strong nanofiller–nanofiller and matrix–nanofiller interactions via H-bonding, as depicted in Scheme 3. The strong interfacial bonding and good interfacial adhesion between GO and PHBHHx as well as with CNC led to the exfoliation of the GO nanosheets. Further, other non-covalent interactions such as polar and hydrophobic can contribute to the development of a reinforcement network firmly stuck to the polymer.

### 4.2. FTIR Analysis

**Figure 2:**
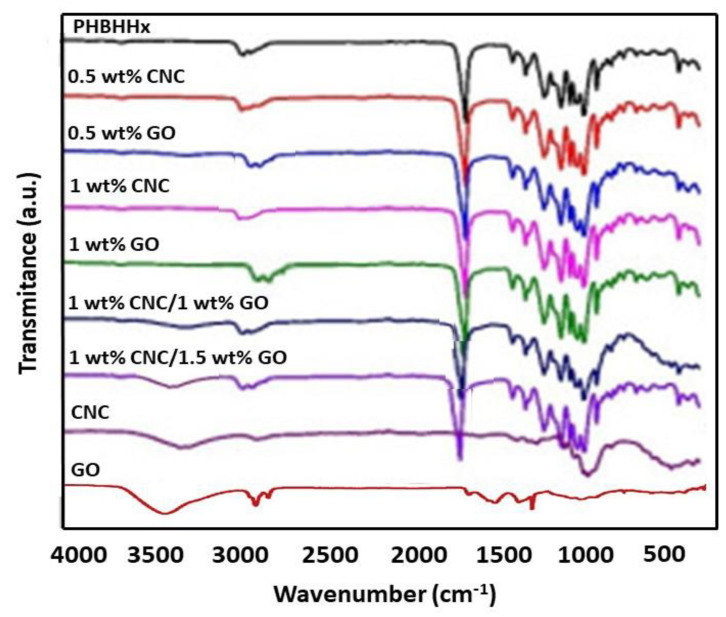
Comparison of FTIR spectrum of neat PHBHHx, CNC, GO, and binary and ternary composites.

To corroborate the presence of the three components in the ternary nanocomposites and obtain some insight into the nanofiller–matrix and nanofiller–nanofiller interactions, the FTIR spectrum of neat PHBHHx, GO, CNC, and its binary and ternary nanocomposites were compared (Figure 2). The raw copolyester shows a very intense peak resulting from the stretching of the COO moieties at 1725 cm^−1^ [23]. The peaks close to 2850 cm^−1^ and 1450 cm^−1^ correspond to the stretching and bending of C–H, respectively. Those between 1300 and 1050 cm^−1^ are related to the stretching of C–O–C moieties, and that at about 1380 cm^−1^ to the bending of the H–C–O, in agreement with results reported earlier [24]. Regarding GO, it comprises carboxylic acids at the boundaries of the sheets as well as hydroxyl and epoxide groups on the two sides of its hexagonal arrangement (Scheme 3). The intense band centered at 3450 cm^−1^ arises from the stretching of O-H groups and that at around 1400 cm^−1^ to the O-H bending. The C-C stretching of aromatic rings appears at 1630 cm^−1^ and the stretching of C-O moieties of the carboxylic acids at around 1700 cm^−1^ [7]. Further, the small band centered at about 1050 cm^−1^ is related to the C-O stretching of the epoxy moieties [25].

In the spectrum of neat CNC, the small peaks at about 2900 and 1650 cm^–1^ correspond to the C–H stretching and the O–H bending vibrations. Additionally, the peak centered at 3400 cm^–1^ is related to the O–H stretching of both hydrogen-bonded and free hydroxyl groups, and the intense band centered at about 1000 cm^−1^ results from the combination of the stretching vibrations of C–C and C–O–C moieties at 1050 and 970 cm^–1^, respectively, corresponding to the crystalline phase of CNC [26]. The spectrum of the nanocomposites is essentially analogous to that of the raw matrix, with some extra small bands arising from the nanofillers like the O-H stretching that can be observed in the ternary nanocomposites shifted to smaller wavenumbers and those broader than that of neat GO or CNC, which could be due to the hydrogen bond formation between the COO moieties of the polymer and the hydroxyl groups of the nanofillers [7]. Further, interactions could also take place between the hydroxyl groups of CNC and carboxylic acid or epoxy moieties of GO, as depicted in Scheme 3. Thus, the interactions between GO and polar molecules comprising hydroxyl groups have been found to result in a broadening of the band arising from the O-H stretching and produce a slight shift towards lower wavenumbers [27]. On the other hand, the band of the carbonyl stretching of PHBHHx appears at a higher wavenumber in the ternary nanocomposites than in the neat polymer (about 10–30 cm^–1^ shift), which could also be attributed to the hydrogen bonding that changed the polarity of the C=O group, ensuing an upshift in the band position. The shift is stronger for the nanocomposite with 1 wt% CNC and 1.5 wt% GO, suggesting the formation of additional hydrogen bonds between the remaining OH moieties of the nanofillers that acted as proton donors and the CO moieties of the ester groups of the polymer that acted as proton acceptors [14]. An analogous phenomenon of a shift in the peak position due to the formation of hydrogen bonds, albeit with smaller changes in wavenumber, has been previously reported for other polyhydroxyalcanoates reinforced with different nanofillers such as ZnO [7], silica [28], or pseudoboehmite [29]. 

### 4.3. XRD Spectra of PHBHHx/CNC/GO Nanocomposites

**Figure 3:**
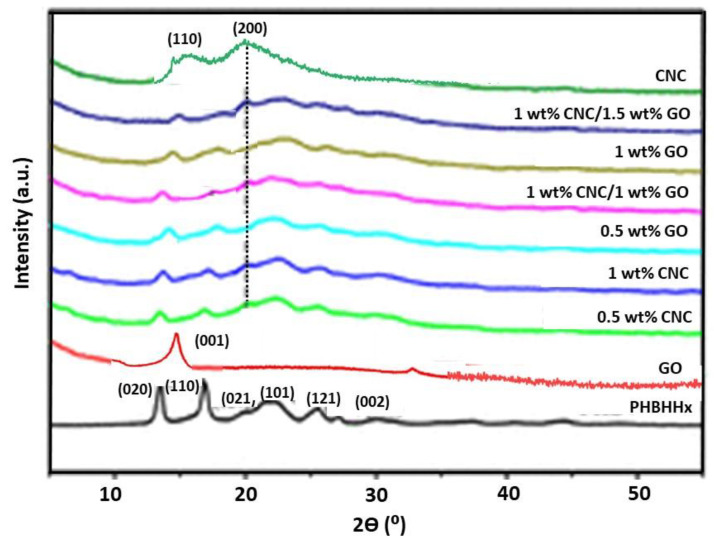
XRD of neat PHBHHx, GO, CNC, and binary and ternary nanocomposites.

XRD analysis was carried out to obtain knowledge about the crystalline nature of the nanocomposites, and the diffractograms of the pure components as well as binary and ternary nanocomposites are compared in Figure 3. The raw copolymer shows diffraction peaks at 2Ɵ of 30.9, 27.5, 25.7, 21.9, 20.3, 17.3, and 13.8°, corresponding to the diffraction of the (002), (040), (121), (101), (021), (110), and (020) planes, respectively. This structure is almost the same as that described for the homopolymer, which is arranged in an orthorhombic unit cell and belongs to the P212121-D2 space group [30]. 

GO exhibits a distinctive peak at 2θ = 14.2°, which is related to the (001) plane. Taking into account Bragg´s law, this corresponds to an interplanar spacing of 0.732 nm and corroborates the random arrangement of the graphene sheets in the GO sample. Further, this peak confirms the successful and complete synthesis of GO from HDFGF, since the diffraction plane in neat graphite appears at 2θ = 26.4° [11]. Regarding CNC, the nanocrystallites are reported to be imperfect [31], thus a significant portion of the nanocellulose structure is referred to as amorphous. In this regard, two strong peaks are found in the XRD of CNC, one at about 16° assigned to the amorphous contribution, and another more intense at about 20° arising from the plane whose Miller index is (200) related to the crystalline phase [32]. 

The patterns of the nanocomposites comprise all the peaks of PHBHHx, demonstrating that the crystalline arrangement of the polymer is retained. Further, the peaks of CNC can also be found, which are stronger in the ternary nanocomposites and shift slightly higher to 2Ɵ. This shift is also indicative of strong CNC-PHBHHx interactions, as discussed earlier. The height of the (020) and (110) diffraction planes of the binary or ternary nanocomposites was notably decreased upon the incorporation of CNC, GO, or both nanofillers into the polymer matrix. This suggests that the crystal growth of PHBHHx in the (110) and (020) planes was hindered in the nanocomposites owing to a strong interaction among the three constituents [14]. It has been described that CNCs speed up the crystallization of polymer matrices, acting as efficient nucleation agents [30], though they do not increase the level of crystallinity or the crystal growth in such directions. Thus, a plausible explanation could be that the presence of both nanofillers leads to two contradictory effects on the polymer crystallization. From one perspective, the nanofillers can behave as nucleating agents that promote the nucleation of polymer crystals and the global speed of the crystallization process. From another perspective, the motion of the PHBHHx segments in some planes could be limited due to the strong nanofiller–polymer chain interactions. However, the chains could preferentially grow along other crystal planes like (101) or (021). Further, in the nanocomposites the diffraction peak arising from the plane (020) was wider than the corresponding peak in the neat polymer, revealing the presence of smaller crystals. Thus, following the Scherrer equation, the crystallite size is inversely proportional to the width of the peaks [33]. It suggests that smaller spherulites were formed, hence the addition of both nanofillers would promote the crystallization of the matrix.

### 4.4. DSC Analysis of the Nanocomposites

**Figure 4:**
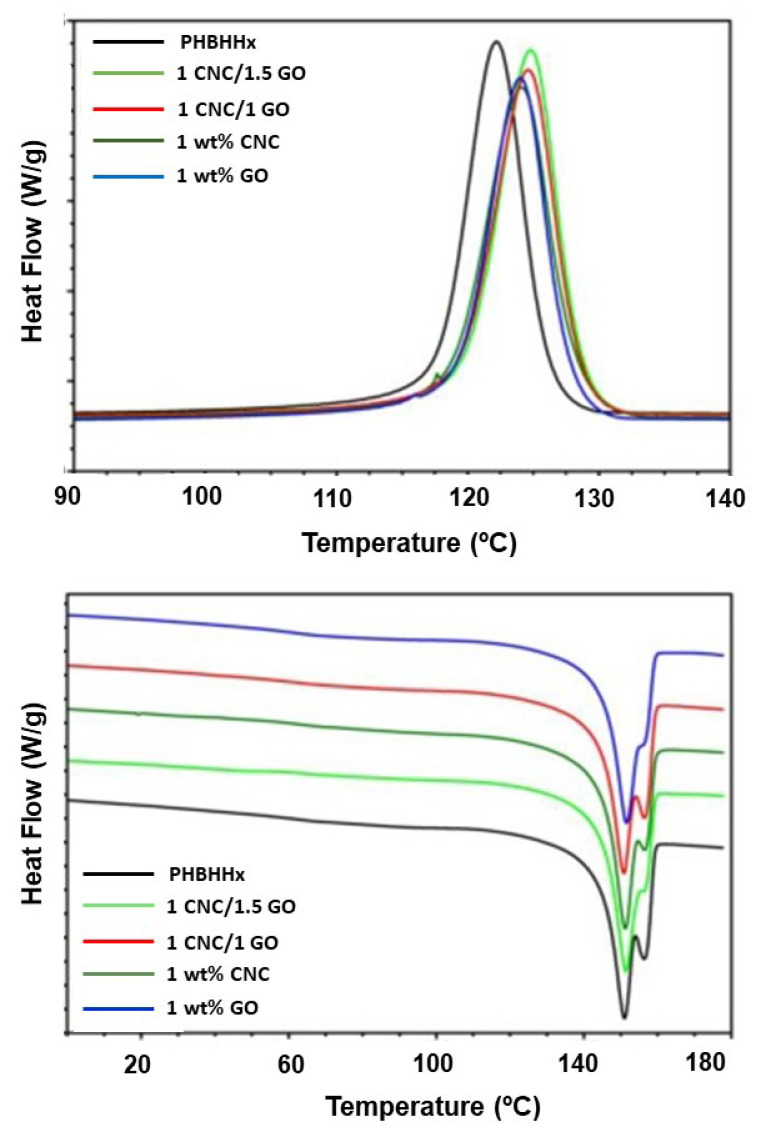
DSC cooling (**top**) and heating (**down**) thermograms of neat PHBHHx and binary and ternary nanocomposites.

DSC experiments were carried out to comparatively assess the effect of the nanofiller type and concentration on the melting and crystallization temperature of PHBHHx, and typical crystallization and heating thermograms of binary and ternary nanocomposites are displayed in Figure 4. The melting and crystallization temperatures (T_m_ and T_c_) and the corresponding enthalpies (ΔH_m_ and ΔH_c_) calculated from the thermograms of all the composites are collected in Table 1. The neat polymer displays dual melting behavior arising from the melting and subsequent re-crystallization and re-melting of the polymer segments throughout heating. The peak appearing at higher temperatures is ascribed to the recrystallization of the matrix [7]. Several thicknesses and levels of the order of the lamellae could be another explanation for the occurrence of two peaks, as well as the presence of segments with diverse orientations and molecular weights [12]. Moreover, compositional heterogeneity in the copolymer could be another cause of the multiple melting phenomenon of PHBHHx. 

Analogous double melting behavior is observed for both binary and ternary nanocomposites, with hardly any modification in the peak positions or the melting enthalpies, in agreement with former reports dealing with polyhydroxyalkanote-based nanocomposites [7,28,30]. Regarding the cooling process, raw PHBHHx displays a T_c_ of about 122 °C, which gradually increases with increasing nanofiller loading, with the higher increment being about 4 °C for the multiscale nanocomposite with 1.5 wt% GO and 1 wt% CNC. These findings support that the nanofillers speed up the crystallization of the polymer via heterogeneous nucleation. Further, both nanofillers seem to have similar nucleating effects, since the T_c_ of the binary nanocomposites is almost identical to that of the ternary nanocomposites with the same total nanofiller loading. This suggests that the nucleation phenomenon is conditioned by the number of nucleating sites, hence by the concentration and not the nanofiller shape. Similar nucleating behavior of PHA crystallization has been described for other nanomaterials such as carbon nanotubes [34], ZnO [7], or SiO_2_ [35], which was ascribed to a uniform nanofiller dispersion and hence results in more sites available for nucleation. Conversely, a drop in T_c_ has been described for composites loaded with nanoclay [36] or NFC [37], ascribed to the compatibility of the PHA with the nanofiller, which hindered the crystallization, or chain decomposition, owing to the remaining humidity in the nanofiber, respectively. Moreover, nanocomposites with ZnO prepared via electrospinning [38] showed lower Tc than the matrix due to the preferential location of the nanofillers in certain regions of the nanofibers. All these facts indicate the impact of the manufacturing route on the nanomaterial distribution, orientation, and position inside the copolymer, hereafter on the ultimate nanocomposite properties.

### 4.5. TGA Analysis of the Nanocomposites

**Figure 5:**
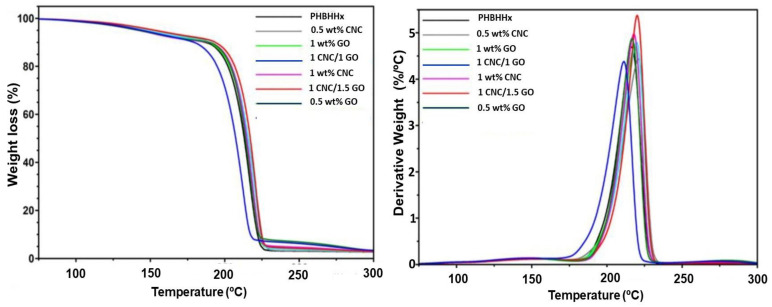
TGA (**left**) and DTG (**right**) thermograms of neat PHBHHx and binary and ternary nanocomposites.

Among the main limitations of polyhydroxyalkanoates is their poor thermal stability, which can result in partial decomposition throughout the processing. Thus, it is crucial to investigate the outcome of nanofillers on the thermal degradation of this copolymer. The TGA and the derivative (DTG) curves in an inert atmosphere of PHBHHx and representative binary and ternary nanocomposites are shown in Figure 5. Further, Table 2 collects the representative degradation temperatures of all the samples tested, i.e., the temperature at 5% weight loss (T_5%_), the onset of degradation (T_onset_), the temperature corresponding to the maximum rate of degradation (T_max_) (maximum of the DTG curve), and the residue at 300 °C (R_300_).

Regarding neat PHBHHx, a single degradation step takes place that begins at ∼204 °C and displays the highest degradation speed at approximately 216 °C (Table 2). The decomposition is believed to progress through a random chain scission mechanism via a cis elimination at the ester linkage of a six-membered ring ester intermediate, also known as the Mclafferty rearrangement [39]. The nanocomposites also exhibit a single degradation stage, though with a small shoulder (as clearly shown in the derivative curves) that increases with increasing the amount of nanofiller, ascribed to the CNC decomposition and/or the elimination of surface-oxygenated groups from GO [40]. Focusing on T_5%_, regarded as a stability measurement during melt processing since it is near the melting point, a gradual rise is found upon increasing nanofiller loading, with the rise being slightly higher for nanocomposites comprising CNC compared with those with the same GO loading. This is surprising since CNC starts to degrade around 330 °C [37], while GO is thermally stable up to 600 °C [25]. This outcome might be attributed to the increased hydrogen bond formation between PHBHHx and CNC compared with GO, which inhibited the random chain scission mechanism and, consequently, improved thermal stability was attained [41]. Accordingly, upon increasing CNC loading, more H-bonds are formed, resulting in improved stability. Analogous behavior of enhanced stability has been reported for other polyhydroxyalkanoates reinforced with carbon nanotubes [34], nanoclay [36], or SiO_2_ [35]. 

An analogous trend was found for T_onset_ and T_max_, with increments up to 6 and 7 °C, respectively, upon the addition of 2.0 wt% CNC. However, a lower onset was detected for binary samples with 0.5, 1, or 1.5 wt% GO compared with the raw polymer. This could be explained considering the fewer functional groups on the GO surface than CNC, hence the limited potential for hydrogen bond formation with the matrix, which induced a slight reduction in this degradation temperature. Further, GO could produce free radicals that promote the decomposition of the polymer chains [42]. However, the multiscale nanocomposites showed significant improvements in all the degradation temperatures. Thus, the nanocomposite with 2.0:0.5 wt% CNC/GO showed the highest T_5%_, T_onset,_ and T_max_ (about 28, 10, and 13 °C higher than those of neat PHBHHx, respectively). This behavior could arise from a synergic phenomenon brought by the formation of hydrogen bonds with the two nanofillers. The homogeneous dispersion of CNC and GO through the copolymer and the strong CNC-GO and PHBHHx–nanofiller interactions through hydrogen bonding likely result in a block effect against the carriage and escape of volatile products generated during the degradation process from the bulk of the matrix to the atmosphere. This effect might delay the development of the ester intermediate, leading to improved thermal stability. Further, the elevated thermal conduction of GO should facilitate the dissipation of heat within the nanocomposite, which is another factor that contributes to the stability enhancement. 

### 4.6. Tensile Properties: Experimental Results

**Figure 6:**
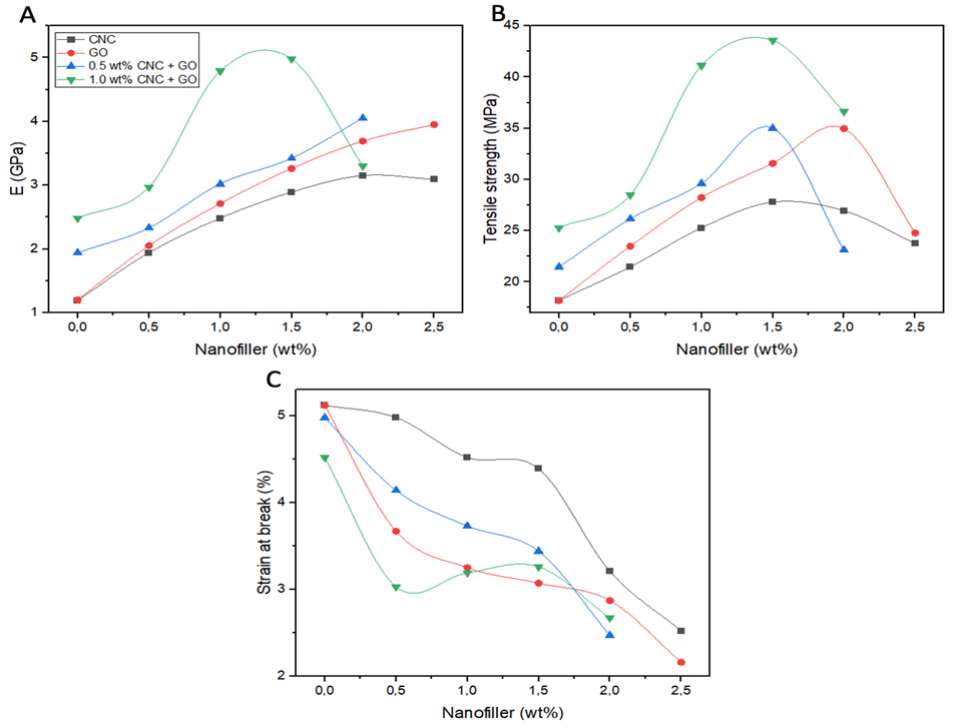
Tensile properties of PHBHHx/CNC/GO nanocomposites versus the total nanofiller content (wt%). The red and black curves indicate nanocomposites filled only with GO or CNC, respectively. The green and blue curves indicate multiscale nanocomposites comprising 1 wt% or 0.5 wt% CNC, respectively, and varying GO loadings. (**A**) Tensile modulus. (**B**) Tensile strength; (**C**) Strain at break.

The results of the tensile tests for all the samples tested are collected in Table 3, and the data are plotted versus the total nanofiller content in Figure 6. The raw copolyester shows a tensile modulus (E) of ∼1.2 GPa, which rises steadily upon growing nanofiller content (Figure 6A), by up to 2.6- and 3.3-fold with the incorporation of 2.5 wt% CNC and GO, respectively. Concerning the composites reinforced solely with CNC, the increase is nearly linear until a concentration of 2.0 wt% and afterward hardly changes, while for those reinforced with GO, the growth is linear in the whole concentration interval investigated. The addition of low contents of 2D flexible GO nanosheets resulted in higher stiffness improvements than the same loadings of 3D rigid CNC with a quasi-spherical shape. This could be related to the increased free volume between spherical nanoparticles compared with nanosheets, which allows more chain mobility, thus resulting in increased deformation [7]. The increments attained in this study are noticeably stronger than those found for polyhydroxyalkanoate-based nanocomposites reinforced with other nanofillers like carbon nanotubes [34], nanoclay [36], or SiO_2_ [35] nanoparticles, signifying that these nanofillers are homogeneously distributed within the copolymer, which hereafter are very effective as nanoreinforcements. Further, the good adhesion between the matrix and nanofiller phases attained by means of hydrogen bonding could also account for the unprecedented stiffness increase found herein. 

Linear growth is also found for the multiscale composites with 0.5 wt% CNC and increasing amounts of GO, up to 3.4-fold for a GO loading of 2.0 wt% (Figure 6A). The intense nanofiller–matrix and nanofiller–nanofiller hydrogen bonding interactions, as stated before, would result in the development of a compact and intercalated network that yields a very strong reinforcing effect. However, for those with 1.0 wt% CNC, E shows the highest value at 1.5 wt% GO and afterwards decreases to some extent, which could be due to a diminution in the matrix–nanocomposite interfacial area. Accordingly, among all the nanocomposites, that with 1 wt% CNC and 1.5 wt% GO shows the greatest modulus (a 4.2-fold increment compared with the value of neat PHBHHx), indicating a synergic effect of the two nanofillers on improving the stiffness of the matrix. The synergistic reinforcing effect of 1:1.5 wt% CNC/GO (blended) on the tensile modulus of PHBHHx was greater than the effect of the same total nanofiller loading of CNC or GO alone (Table 3). 

A different behavior can be observed for the tensile strength (Figure 6B). This property increases up to 1.5 wt% CNC or 2.0 wt% GO and then decreases slightly. This could be elucidated taking into account that in the samples with higher CNC or GO loading, the 3D quasi-spherical nanoparticles or the 2D nanosheets confine the ductile deformation of the matrix chains, as illustrated in Figure 6C, resulting in early failure, which in turn leads to poorer strength. This could arise from the strong hydrogen bonding interactions between the polymer and CNC or GO via hydroxyl groups. In the case of the ternary nanocomposites, a maximum in strength is found for the nanocomposite with 1 wt% CNC and 1.5 wt% GO, similarly to the tensile modulus, the maximum increment being about 2.4-fold compared with the neat copolymer. Focusing on the elongation at break, a decreasing trend is systematically found upon growing nanofiller loading. In the binary nanocomposites, the decrease is more pronounced for those with GO compared with the same CNC loading, despite GO having less hydroxyl surface groups. The 2D shape of this nanofiller could impose stronger restrictions on polymer chain mobility, hence the drop in ductility is more pronounced (about 2.3-fold for a GO loading of 2.5 wt%). Concerning the multiscale hybrids with 0.5 wt% CNC and growing concentrations of GO, the drop in ductility is less pronounced than upon the addition of GO alone. The CNC likely intercalates between the nanolayers of GO, resulting in a more disentangled structure with a huge contact area with the copolymer, henceforth creating an intimate adhesion between the phases, which in turn results in a slighter drop in this parameter. Conversely, those incorporating 1.0 wt% CNC and growing loadings of GO show a stronger drop in ductility. Overall, the hybrid nanocomposite with 1.5 wt% GO and 1.0 wt% CNC shows the optimal balance of mechanical properties. 

### 4.7. Prediction of Mechanical Properties Using the SVM Model

The SVM model was chosen herein to predict the tensile properties of the hybrid composites since it has the skill to handle non-linear data and to deal with big data sets, as well as to recognize patterns and generalize, thus enhancing the accuracy of the predictions. SVM demonstrates similar or even better performance than other ML models such as neural networks (NNs) to solve real, current challenges and issues [43,44]. The chief aim of support vector regression (SVR) is to attain a function that approximates the relation between the target variables and the input data. Further, errors are not decisive if they are smaller than a specified value [43]. That is, the main idea is to find the best-fitting line. 

The predictions of the tensile properties (modulus, strength, and elongation at break) were made herein using the SVR algorithm in combination with the hyperparameter search technique RandomSearchCV, where the values of C and epsilon are varied (Table 4). From the available data, 80% of the data is used for training, 15% for testing, and 5% for evaluation.

Regarding the tensile modulus, *R*^2^ of the training set was found to increase by increasing the value of the C parameter and keeping epsilon constant, whilst *R*^2^ of the test set decreased. This shows that the model better describes the training data as C increases, but there may be an increased risk of overfitting, resulting in underperformance on unseen data.

On the other hand, for a constant C value of 10, a rise in epsilon results in a growth in *R*^2^ of the training set. However, the accuracy of the test set remains almost unchanged or even slightly decreases. This suggests that a higher epsilon value allows for a better fit to the training data, though it does not significantly improve the ability of the model to generalize to unseen data.

To achieve an optimal balance between performance on the training set and the test set, a model is sought that fits the training data well and generalizes effectively on unseen data. The combination of C = 8 and epsilon = 0.03 shows good performance on both sets, with an *R*^2^ of 0.935 on the training set and an *R*^2^ of 0.723 on the test set (Table 4). This combination can be considered as an option that seeks to strike a balance between model fit and generalization. However, it is important to consider different evaluation metrics (MSE and MAE) before making a final decision on the optimal combination of parameters to achieve the desired balance.

Focusing on the tensile strength (Table 4), it is found that the *R*^2^ of the training set hardly changes while *R*^2^ of the test set decreases with increasing epsilon when the value of C is set constant. On the other hand, *R*^2^ of the training set increases by increasing the value of C and keeping epsilon constant. This indicates that the training data are better fitted by the model. However, the accuracy of the test set is simultaneously reduced, suggesting that the model may be overfitting the training data and is not generalizing effectively to new data. The combination of C = 40 and epsilon = 0.4 leads to a reasonable balance between model fit and generalizability for both the training (*R*^2^ = 0.881) and the test set (*R*^2^ = 0.792).

Regarding the elongation at break, in general, all parameter combinations show good performance in terms of *R*^2^ in both the training and test sets. However, additional patterns and trends are identified when the value of epsilon is modified while C is kept constant. Variations in the epsilon parameter have a noteworthy effect on the model performance and influence its generalizability; hence, it is a relevant parameter to consider. For example, epsilon values of 0.06 and 0.08 for a constant value of C = 100 led to different *R*^2^ values in both sets (Table 4). On the other hand, no clear relationship is found between the C value and *R*^2^ values in the training and test sets. No obvious trend can be identified regarding the influence of C on the model performance for this property. Overall, the combinations with a constant epsilon value of 0.1 and C values of 300 and 400 show an optimal balance between the training and the test set, with good performance on both sets. 

Table 5 summarizes *R*^2^, MSE, and MAE values for the combinations selected as the optimum for the prediction of each mechanical property. The results indicate that the SVR model is well-suited to predict the mechanical properties of ternary PHBHHx/CNC/GO nanocomposites. The most accurate prediction (highest *R*^2^) corresponds to the elongation at break, whilst less efficient prediction is obtained for the tensile strength, which might be explained considering that this property did not show a direct dependence nor a clear tendency with the nanofiller content, as displayed in Figure 6B. Thus, the nanocomposite stiffness is highly dependent on the modulus of each component, whereas the strength depends also strongly on additional parameters, such as the orientation and level of nanomaterial dispersion as well as the interfacial adhesion between the components; consequently, it is more challenging to predict the strength of the hybrids from the values of each of the constituents and their weight percentages. Nonetheless, other factors should be taken into account and considered for further analysis, such as the evaluation of other metrics to fully understand the model performance.

MSE and MAE metrics were used to evaluate the accuracy and level of error in the predictions made by the SVR model. MSE is considered one of the crucial validation criteria of ML models. Low values of MSE and MAE indicate that the model is able to make accurate predictions. The smaller the MSE and MAE values, the closer the predicted and the experimental values. Data in Table 5 indicate that the SVR model has a promising performance in terms of accuracy, as it shows low levels of error in the predictions of the evaluated mechanical properties. These errors were smaller than those described before for the prediction of the tensile properties of nanocomposites using ML models (i.e., MAE values close to 3 [45] or even greater, around 4.2 [46]). Again, the smallest MSE and MAE correspond to the elongation at break, while the highest to the tensile strength. Overall, it was found that the SVR model has great potential for modeling the mechanical properties of multiscale polymer-based nanocomposites.

## 5. Conclusions

Ternary bionanocomposites based on PHBHHx copolymer reinforced with different loadings of CNC and GO have been prepared through an easy and environmentally friendly tactic based on the solution casting method. SEM analysis corroborated the uniform and random distribution of both fillers throughout the polymer. FTIR confirmed the presence of the three components in the nanocomposites and suggested the formation of hydrogen bonds between them, which hindered the crystal growth of the polymer, as evidenced by the results of DSC and XRD analyses. The nanofillers had a heterogenous nucleating effect, increasing the crystallization temperature of the polymer. They also increased the thermal stability, which was ascribed to a synergic block effect of both nanofillers that hampers the carriage of the decomposition products from the bulk of the nanocomposite to the gas phase. Further, unprecedented increments in stiffness and strength were found upon incorporation of the two nanomaterials, which were attributed to a synergistic reinforcing effect. Thus, the composite with 1.5 wt% GO and 1.0 wt% CNC shows the optimal balance of properties. The mechanical properties for various concentrations of CNC and GO were accurately predicted using the SVR model. The model performance was evaluated in terms of the correlation coefficient (*R*^2^), mean absolute error (MAE), and mean square error (MSE). The highest *R*^2^ and the smallest MSE and MAE correspond to the elongation at break, while the lowest *R*^2^ and the highest MSE and MAE correspond to the tensile strength. These bionanocomposites open novel opportunities for future developments, mainly in the medical and food packaging fields.

## Figures and Tables

**Table 1:** DSC parameters for neat PHBHHx and binary and ternary nanocomposites. ΔHm: melting enthalpy; ΔH_c_: crystallization enthalpy; T_m_: melting temperature; and T_c_: crystallization temperature.

Nanofiller Loading	T_m1_/T_m2_ °C	ΔH_m1_/ΔH_m2_ J/g	T_c_ °C	ΔH_c_ J/g
0	153.8/160.3	57.8/18.3	121.8	73.9
0.5 wt% CNC	153.9/160.5	56.5/17.2	122.7	73.7
1.0 wt% CNC	153.6/161.2	56.8/16.9	123.8	73.5
1.5 wt% CNC	154.2/161.6	56.3/17.1	124.6	74.2
2.0 wt% CNC	154.7/161.5	56.9/16.5	125.7	74.0
0.5 wt% GO	153.6/159.7	56.2/18.2	122.9	73.9
1.0 wt% GO	154.4/159.0	60.3/10.9	124.1	69.8
1.5 wt% GO	154.7/161.8	56.5/18.3	124.8	74.5
2.0 wt% GO	154.6/161.3	56.2/17.9	125.5	74.2
0.5 wt% CNC/0.5 wt% GO	154.5/159.1	60.4/10.7	124.2	69.7
1 wt% CNC/1 wt% GO	153.6/160.5	61.3/14.5	124.5	73.8
1 wt% CNC/1.5 wt% GO	154.2/159.2	63.1/12.1	125.4	75.1
1.5 wt% CNC/1.0 wt% GO	154.7/159.9	63.4/12.3	125.1	75.2
2.0 wt% CNC/0.5 wt% GO	154.5/160.2	63.2/12.5	125.3	74.9
0.5 wt% CNC/2.0 wt% GO	154.9/160.1	63.4/12.7	124.9	74.7

**Table 2:** TGA data for neat PHBHHx and binary and ternary nanocomposites. T_5%_: temperature at 5% weight loss; T_onset_: temperature corresponding to the beginning of degradation; T_max_: temperature of the maximum rate of degradation; R_300_: residue at 300 °C.

Nanofiller Loading	T_5%_ (°C)	T_onset_ (°C)	T_max_ (°C)	R_300_ (%)
0	141.9	204.1	216.3	2.25
0.5 wt% CNC	148.5	206.5	220.6	2.28
1.0 wt% CNC	151.2	208.7	221.5	2.34
1.5 wt% CNC	154.9	209.7	222.3	2.54
2.0 wt% CNC	156.7	209.8	222.9	2.76
0.5 wt% GO	142.6	199.6	211.6	2.40
1.0 wt% GO	146.7	201.7	218.7	2.52
1.5 wt% GO	147.6	203.4	219.6	2.59
2.0 wt% GO	150.0	205.6	220.5	2.66
0.5 wt% CNC/0.5 wt% GO	153.5	209.0	222.1	2.56
1 wt% CNC/1 wt% GO	162.5	211.5	224.0	2.64
1 wt% CNC/1.5 wt% GO	168.2	213.7	226.7	2.82
1.5 wt% CNC/1.0 wt% GO	165.4	212.4	225.9	2.68
2.0 wt% CNC/0.5 wt% GO	169.9	214.5	228.9	2.95
0.5 wt% CNC/2.0 wt% GO	166.4	212.9	226.0	2.88

**Table 3:** Tensile properties of PHBHHx/CNC/GO hybrid composites with different concentrations of CNC and GO. For comparison, data of binary composites are also included.

Nanofiller Loading	Tensile Modulus (GPa)	Tensile Strength (MPa)	Strain at Break (%)
0	1.19	18.18	5.12
0.5 wt% CNC	1.94	21.46	4.98
1.0 wt% CNC	2.48	25.27	4.52
1.5 wt% CNC	2.89	27.79	4.39
2.0 wt% CNC	3.15	26.94	3.21
2.5 wt% CNC	3.09	23.75	2.52
0.5 wt% GO	2.05	23.48	3.67
1.0 wt% GO	2.71	28.23	3.25
1.5 wt% GO	3.26	31.58	3.07
2.0 wt% GO	3.69	34.97	2.87
2.5 wt% GO	3.95	24.78	2.16
0.5 wt% CNC/0.5 wt% GO	2.33	26.15	4.14
0.5 wt% CNC/1.0 wt% GO	3.02	29.61	3.73
0.5 wt% CNC/1.5 wt% GO	3.42	35.00	3.44
0.5 wt% CNC/2.0 wt% GO	4.05	23.12	2.47
1.0 wt% CNC/0.5 wt% GO	2.67	28.48	3.03
1.0 wt% CNC/1.0 wt% GO	4.79	41.12	3.19
1.0 wt% CNC/1.5 wt% GO	4.98	43.57	3.26
1.0 wt% CNC/2.0 wt% GO	3.30	36.63	2.67
1.5 wt% CNC/0.5 wt% GO	4.23	32.36	2.59
2.0 wt% CNC/0.5 wt% GO	3.51	25.47	2.23
1.5 wt% CNC/1.0 wt% GO	4.25	35.74	3.56

**Table 4:** *R*^2^ values of the training and test sets for predicting the mechanical properties of the nanocomposites for different values of epsilon and C hyperparameters of the SVM model.

Property	C	Epsilon	*R*^2^ Training	*R*^2^ Test
Tensile modulus	6	0.03	0.922	0.742
Tensile modulus	8	0.03	0.935	0.723
Tensile modulus	10	0.05	0.938	0.709
Tensile modulus	10	0.08	0.941	0.703
Tensile strength	40	0.4	0.881	0.792
Tensile strength	40	0.7	0.881	0.786
Tensile strength	30	0.6	0.706	0.938
Tensile strength	50	0.6	0.909	0.751
Elongation at break	300	0.1	0.982	0.818
Elongation at break	400	0.1	0.982	0.818
Elongation at break	100	0.06	0.993	0.812
Elongation at break	100	0.08	0.988	0.821

**Table 5:** Summary of *R*^2^, MSE, and MAE for the best combinations of hyperparameters in SVR for mechanical properties prediction.

Property	*R*^2^ Training	*R*^2^ Test	MSE	MAE
Tensile modulus	0.935	0.723	0.1932	0.3677
Tensile strength	0.881	0.792	0.8149	1.2001
Elongation at break	0.982	0.818	0.1568	0.2781

## Data Availability

Data will be available on request.

## Supplementary Materials

The following supporting information can be downloaded at: https://www.mdpi.com/article/10.3390/polym15183746/s1, Figure S1: Top: SEM (a) and TEM (b) image of the as-synthetized CNC. Bottom: SEM (c) and TEM (d) image of raw GO. Figure S2. Photographs of PHBHHx nanocomposites with (a) 1 wt% GO; (b) 1 wt% CNC; (c) 1 wt% CNC; and 1.5 wt% GO.
