# Supplementary material for: Poly(3-hydroxybutyrate-co-3-hydroxyhexanoate) Bionanocomposites with Crystalline Nanocellulose and Graphene Oxide: Experimental Results and Support Vector Machine Modeling
Source: Polymers (Basel). 2023 Sep 13;15(18):3746. doi: 10.3390/polym15183746 (PMC10537444; doi:10.3390/polym15183746)
Supplement: Supplementary file 1 [file polymers-15-03746-s001.zip › polymers-2570347-supplementary.pdf]

Supplementary Materials

Poly(3-hydroxybutyrate-co-3-hydroxyhexanoate) Bionanocomposites with Crystalline Nanocellulose and Graphene Oxide: Experimental Results and Support Vector Machine Modeling

Elizabeth Champa-Bujaico [1], Ana M. Díez-Pascual [2,*] and Pilar Garcia-Diaz [1]

[1] Departamento de Teoría de la Señal y Comunicaciones, Universidad de Alcalá, Ctra. Madrid-Barcelona Km. 33.6, 28805 Alcalá de Henares, Madrid, Spain
[2] Facultad de Ciencias, Departamento de Química Analítica, Química Física e Ingeniería Química, Universidad de Alcalá, Ctra. Madrid-Barcelona, Km. 33.6, 28805 Alcalá de Henares, Madrid, Spain
* Correspondence: am.diez@uah.es

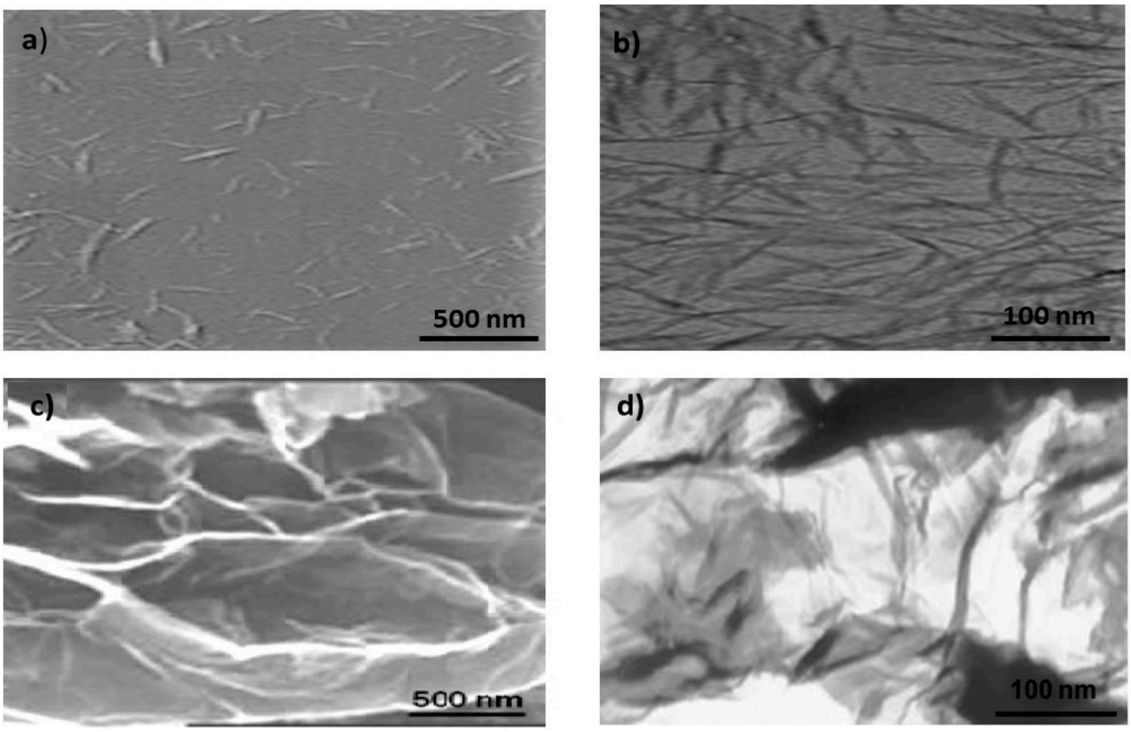

Figure S1. Top: SEM (a) and TEM (b) image of the as-synthetized CNC. Bottom: SEM (c) and TEM (d) image of raw GO.

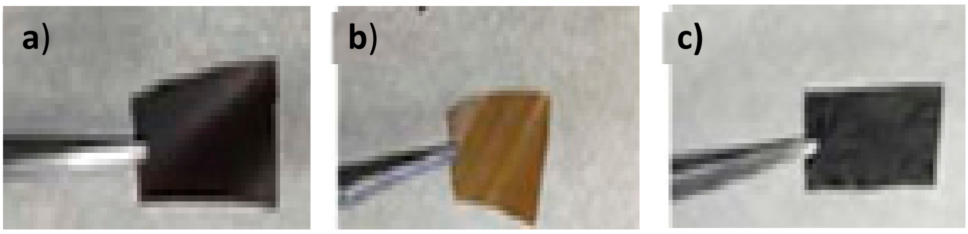

Figure S2. Photograhs of PHBHHx nanocomposites with (a) 1 wt% GO; (b) 1 wt% CNC; (c) 1 wt% CNC and 1.5 wt% GO
